# Targeting the adaptive molecular landscape of castration-resistant prostate cancer

**DOI:** 10.15252/emmm.201303701

**Published:** 2015-04-20

**Authors:** Alexander W Wyatt, Martin E Gleave

**Affiliations:** Vancouver Prostate Centre & Department of Urologic Sciences, University of British ColumbiaVancouver, BC, Canada

**Keywords:** androgen receptor, castration-resistant prostate cancer, stress response, survival pathways, tumour heterogeneity

## Abstract

Castration and androgen receptor (AR) pathway inhibitors induce profound and sustained responses in advanced prostate cancer. However, the inevitable recurrence is associated with reactivation of the AR and progression to a more aggressive phenotype termed castration-resistant prostate cancer (CRPC). AR reactivation can occur directly through genomic modification of the AR gene, or indirectly via co-factor and co-chaperone deregulation. This mechanistic heterogeneity is further complicated by the stress-driven induction of a myriad of overlapping cellular survival pathways. In this review, we describe the heterogeneous and evolvable molecular landscape of CRPC and explore recent successes and failures of therapeutic strategies designed to target AR reactivation and adaptive survival pathways. We also discuss exciting areas of burgeoning anti-tumour research, and their potential to improve the survival and management of patients with CRPC.

## Introduction

Normal prostate epithelial cells become malignant through deregulation to context-specific tumour suppressors and oncogenes (Taylor *et al*, 2010; Barbieri *et al*, 2012; Baca *et al*, 2013). Although the precise combination of genomic aberrations is notoriously heterogeneous between patients (Wyatt *et al*, 2014), at diagnosis the most common events include rearrangements affecting ETS gene family members, mutations in the ubiquitin ligase SPOP, disruption to the PI3K antagonist PTEN and copy number gain of oncogenic transcription factor MYC (Taylor *et al*, 2010; Barbieri *et al*, 2012). However, true to its hormone-regulated non-malignant ancestor, prostate cancer cells remain dependent on a ligand-activated androgen receptor (AR) to facilitate mitogenic responses enabled by genomic aberration. Despite enormous genomic heterogeneity, this biological homogeneity means that almost all tumours will initially respond to ligand depletion of the AR. Consequently, surgical or chemical castration, and subsequent elimination of most circulating testosterone, has been a mainstay of prostate cancer treatment for over 70 years (Huggins *et al*, 1941).

Unfortunately, due again to an innate ancestral ability, castration induces adaptive stress responses in prostate cancer cells that insulates against apoptosis. This, together with the steady accrual of further genomic and epigenomic aberration (Grasso *et al*, 2012), facilitates inevitable progression to a more aggressive tumour capable of growing in castrate levels of testosterone and thus termed castration-resistant prostate cancer (CRPC). The majority of CRPC re-initiate mitogenesis by reactivation of the AR: meaning that analogous targeting of the AR signalling axis is efficacious, at least for a short duration, in a large proportion of CRPC patients (Fizazi *et al*, 2012; Scher *et al*, 2012; Ferraldeschi *et al*, 2015). The precise nature and speed of AR reactivation is governed by existing and accruing genomic aberration, and with increasing cycles of AR axis targeting, this aberration can become more critical in enabling growth and converting innate adaptive survival responses into hard-wired assets. In fact, in the modern era of effective AR axis inhibition at different stages of progression, it is now relatively common to find late-stage CRPC where the AR has become incidental to a tumour's successful growth and evolution (Beltran *et al*, 2012; Aparicio *et al*, 2013; Pezaro *et al*, 2014a).

Until 2010, only the cytotoxic docetaxel had demonstrated a clear survival benefit in patients progressing on first-line androgen deprivation therapy and first-generation AR antagonists (compounds that compete with endogenous ligand binding the AR) (Tannock *et al*, 2004). However, the revelation of AR reactivation in CRPC led to development of a wave of agents designed to better inhibit the AR signalling axis, with abiraterone acetate and enzalutamide the first to be approved (Fizazi *et al*, 2012; Scher *et al*, 2012). This review explores adaptive survival responses and genomic aberrations that facilitate AR reactivation in CRPC, and the recent successes and failures of strategies designed to exploit such changes. We describe the evolving landscape of treatment resistance that is developing in response to new agents, and discuss novel targets of therapeutic potential. Finally, we highlight the need for predictive biomarkers and evaluate the promise of liquid biopsies to help guide development and implementation of emerging therapeutics.

## Direct reactivation of the androgen receptor

### Renewed androgen synthesis in the castrate setting

Despite castrate levels of circulating testosterone, CRPC tissue often has higher levels of intra-tumoural androgens than non-CRPC counterparts, implying restoration of ligand for the AR (Mostaghel *et al*, 2007; Montgomery *et al*, 2008). This is due in part to an innate feedback mechanism inherited from non-malignant prostate epithelial cells, enabling them to adapt to varying levels of steroid. Indeed, a suite of steroidogenic enzymes is epigenetically up-regulated in CRPC (Stanbrough *et al*, 2006; Mitsiades *et al*, 2012), particularly those resulting in accumulation of dihydrotestosterone (DHT, the testosterone metabolite preferred by the AR). This expression program can leverage adrenal androgens (Montgomery *et al*, 2008), or even initiate *de novo* synthesis of testosterone (Locke *et al*, 2008). At the extreme end of the spectrum are tumours with mutations in HSD3B1 (an enzyme governing a rate-limiting step in DHT synthesis) that facilitate accumulation of protein and hence increased DHT synthesis (Chang *et al*, 2013).

**Figure 1:**
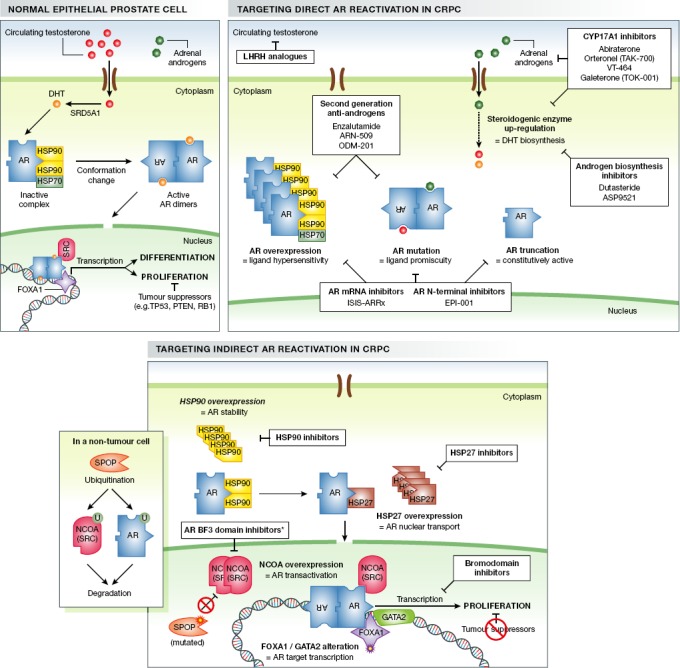
Mechanisms of androgen receptor reactivation in castration-resistant prostate cancer The top left panel depicts the activation of the androgen receptor (AR) by its natural ligand (dihydrotestosterone, DHT) in a normal cell. Induction of functional tumour suppressors prevents the AR transcriptional program from driving mitogenesis. The top right panel shows adaptive and genomic changes in CRPC cells that can lead to direct reactivation of the AR in the absence of natural ligand. White boxes illustrate novel agents (targeted against AR reactivation) recently approved or currently undergoing clinical evaluation for the treatment of CRPC. The bottom panel demonstrates the contribution of AR co-factors and co-chaperones to the reactivation of AR in CRPC and illustrates novel targeting strategies in development.

Ultimately, testosterone and DHT synthesis are dependent on catalytic conversion of cholesterol by members of cytochrome P450 (CYP) family of enzymes. CYP17A1 is a pivotal enzyme in this process, required for both canonical and alternative androgen synthesis, and has consequently been the focus of concerted drug development over the past decade (Fig1). This strategy was vindicated when phase III trials of the CYP17A1 inhibitor abiraterone acetate in metastatic CRPC (mCRPC) patients post- and pre-chemotherapy demonstrated an improved median overall survival (Fizazi *et al*, 2012; Ryan *et al*, 2013). The success of abiraterone has accelerated development of other CYP17A1 inhibitors, particularly compounds that specifically inhibit the 17,20-lyase activity of CYP17A1 and thereby render glucocorticoid co-admission unnecessary (Ferraldeschi *et al*, 2013). One such agent, orteronel (TAK-700; Kaku *et al*, 2011), was recently evaluated in two large phase III trials in mCRPC patients pre- and post-chemotherapy (NCT01193244; NCT01193257), but failed to demonstrate an overall survival benefit (Dreicer *et al*, 2014; Saad *et al*, 2015). These trials were likely confounded by post-study availability of abiraterone, and moreover, since both trials co-administered orteronel with prednisone, its vaunted 17,20-lyase specificity was not exploited. Two further next-generation CYP17A1 inhibitors, VT-464 (Toren *et al*, 2015) and galeterone (TOK-001) (Handratta *et al*, 2005) are currently undergoing phase I and II development, respectively (NCT02012920; NCT01709734) (Table1). VT-464 demonstrated anti-cancer activity in preclinical models of advanced CRPC, significantly lowering tumoural androgen levels in castrate mice, and enforcing greater suppression of the AR signalling axis compared to abiraterone (Toren *et al*, 2015). Galeterone showed promising phase I activity in chemotherapy-naïve CRPC patients (Taplin *et al*, 2012) and has the convenient side-effect of AR cross-inhibition.

**Table 1:** Selected ongoing clinical trials for novel treatments of patients with CRPC

Agent(s)	Activity	Phase	Trial ID
Targeting the AR axis			
VT-464	Lyase-selective inhibitor of CYP17	I/II	NCT02012920
Galeterone (TOK-001)	Dual CYP17 inhibitor and AR antagonist	II	NCT01709734
ARN-509	Second-generation AR antagonist	III (SPARTAN)	NCT01946204
ODM-201	Second-generation AR antagonist	III (ARAMIS)	NCT02200614
Enzalutamide + abiraterone	Second-generation AR antagonist; CYP17 inhibitor	II	NCT01650194
ARN-509 + abiraterone	Second-generation AR antagonist; CYP17 inhibitor	Ib	NCT01792687
Enzalutamide ± abiraterone	Second-generation AR antagonist: CYP17 inhibitor	III	NCT01949337
Targeting adaptive survival pathways			
OGX-427 + abiraterone	HSP27 inhibitor; CYP17 inhibitor	II	NCT01681433
AT13387 + abiraterone	HSP90 inhibitor; CYP17 inhibitor	I/II	NCT01685268
GDC-0068 + abiraterone	Pan AKT inhibitor; CYP17 inhibitor	Ib/II	NCT01485861
BEZ235 + abiraterone	Dual PI3K and mTOR inhibitor; CYP17 inhibitor	Ib	NCT01634061
BKM120 + abiraterone	Pan PI3K inhibitor; CYP17 inhibitor	Ib	NCT01634061
AZD8186	PI3K beta and delta inhibitor	I	NCT01884285
GSK2636771	PI3K beta inhibitor	I/IIa	NCT01458067
Cabozantinib + abiraterone	Tyrosine kinase inhibitor; CYP17 inhibitor	I	NCT01574937
Dasatinib ± abiraterone	Tyrosine kinase inhibitor; CYP17 inhibitor	II (randomized)	NCT01685125
Sunitinib or dasatinib ± abiraterone	Tyrosine kinase inhibitors; CYP17 inhibitor	II (randomized)	NCT01254864
Tivozanib + enzalutamide	VEGF inhibitor; Second-generation AR antagonist	II	NCT01885949
Dovitinib + abiraterone	Tyrosine kinase inhibitor; CYP17 inhibitor	II	NCT01994590
OGX-011 ± cabazitaxel	CLU inhibitor; microtubule inhibitor	III (AFFINITY)	NCT01578655
Alisertib + abiraterone	AURKA inhibitor; CYP17 inhibitor	I/II (randomized)	NCT01848067
Inhibiting DNA repair			
Olaparib	Selective PARP1 inhibitor	II	NCT01682772
Veliparib ± abiraterone	PARP inhibitor	II (randomized)	NCT01576172
Immunotherapy			
PROSTVAC		III (Prospect)	NCT01322490
Targeting neuroendocrine prostate cancer			
Alisertib (MLN8237)	AURKA inhibitor	II	NCT01799278

Although CYP17A1 is a critical hub in steroidogenesis, there is a clear rationale to co-target other members in the pathway. Indeed, dutasteride, which inhibits 5-alpha-reductase (SRD5A1) catalysis of testosterone to DHT, is currently being tested in combination with abiraterone (NCT01393730). Similarly, AKR1C3 is a promising target given its significant up-regulation in CRPC and key role reducing androstenedione to testosterone (Adeniji *et al*, 2013). Although the selective oral AKR1C3 inhibitor ASP9521 (Kikuchi *et al*, 2014) showed no response in phase I/II trials in mCRPC (Loriot *et al*, 2014), it likely requires use in combination given the alternative pathways to generate DHT.

### Genomic modification of the androgen receptor

Even in a castrate-sensitive cell, ligand depletion triggers an innate feedback response leading to increased transcription of the AR gene (Wolf *et al*, 1993; Cai *et al*, 2011; Knuuttila *et al*, 2014; Wyatt *et al*, 2014). The consequent overexpression of AR in CRPC tissue confers hypersensitivity to low levels of androgen as well as facilitating antagonist to agonist conversion for some first-generation AR antagonists (Chen *et al*, 2004). In over 60% of initial CRPC, AR overexpression is driven by X chromosome rearrangement and subsequent focal copy number gain (Grasso *et al*, 2012). The persistent transcriptional pressure on the AR gene caused by ligand depletion probably confers susceptibility to DNA breakage (Mathas & Misteli, 2009) and is likely to be partly responsible for AR amplification.

Therefore, in parallel to the clinical development of CYP17A1 inhibitors, awareness of AR up-regulation in CRPC led to wave of second-generation AR antagonists that compete more effectively with androgen for the ligand-binding domain (LBD) of the AR (Fig1). The first to enter the clinic was enzalutamide, conferring an overall survival improvement for both post- and pre-chemotherapy CRPC patients in phase III trials (Scher *et al*, 2012; Beer *et al*, 2014). The analogous compound ARN-509 has showed encouraging activity in phase I trials in chemotherapy-naïve CRPC patients before and after abiraterone treatment (Rathkopf *et al*, 2013b) and is currently under phase III evaluation in non-metastatic CRPC (NCT01946204), with a primary endpoint of delay in progression to M1 disease. A third potent AR antagonist, ODM-201, has a distinctly different structure than enzalutamide or ARN-509 and having shown promise in recent phase I/II trials (Fizazi *et al*, 2014), will now be evaluated in a phase III trial in men with high-risk non-metastatic CRPC (NCT02200614). The success of these new AR-LBD antagonists reflects more potent inhibition of DHT binding to the AR and prevention of AR nuclear translocation and binding to target promoters (Tran *et al*, 2009; Clegg *et al*, 2012).

This increasing arsenal of more potent anti-AR drugs will provide more tools to combat the non-synonymous AR mutations that are detectable in 10–20% of initial CRPC patients (Grasso *et al*, 2012; Beltran *et al*, 2013). The majority of documented mutations fall within the LBD or cofactor binding regions (Gottlieb *et al*, 2012), reducing binding specificity and permitting activation of the AR by adrenal androgens or other steroid metabolites. Certain LBD mutations are also sufficient to convert AR antagonists to AR agonists and are likely responsible for the 15–30% of patients that exhibit a withdrawal syndrome after cessation of first-generation therapies (e.g. bicalutamide, flutamide) (Small *et al*, 2004). However, since individual mutations do not tend to confer pan-antagonist resistance, the development of real-time strategies to monitor for AR mutation emergence could guide rational sequencing of AR pathway inhibitors. Interestingly, although a recently reported AR mutation (F876L) can drive resistance to both enzalutamide and ARN-509 *in vitro* (Balbas *et al*, 2013; Joseph *et al*, 2013; Korpal *et al*, 2013), few cases of enzalutamide withdrawal syndrome have been reported to date (Rodriguez-Vida *et al*, 2015). Optimistically, this may suggest conversion of enzalutamide from antagonist to agonist is rare, but it may simply reflect that the contemporary metastatic landscape can have multiple independent tumour clones, each responding differently to therapy (Carreira *et al*, 2014). AR mutations will also be a mechanism of resistance to CYP17A1 inhibitors, especially agents requiring prednisone co-admission, since certain mutations (e.g. L702H) can repurpose glucocorticoids as AR ligand (Carreira *et al*, 2014). Furthermore, since abiraterone increases progesterone levels and appears to select for the progesterone-activating mutation T877A (Chen *et al*, 2015), promiscuous AR proteins in general are likely to continue to confound DHT depletion strategies.

Unfortunately, any series of agents competing in a similar therapeutic space will encounter cross-resistance. In CRPC, cycles of increasingly potent AR therapeutics augment the selective pressure for AR aberration, which can drive primary resistance to the next agent in line. Drug sequence strategies have been further compounded by suggestion that part of the activity of docetaxel in CRPC patients can be attributed to a microtubule-dependent effect on AR activity (Gan *et al*, 2009; Zhu *et al*, 2010; Darshan *et al*, 2011). Indeed, the activity of docetaxel is significantly reduced after abiraterone treatment (Mezynski *et al*, 2012), although this effect may not be due to cross-resistance *per se* (Azad *et al*, 2014; de Leeuw *et al*, 2015). Early evidence also suggests that enzalutamide elicits only a modest response rate in the post-docetaxel and post-abiraterone population (Bianchini *et al*, 2014; Schrader *et al*, 2014; Azad *et al*, 2015a) and equally and that abiraterone treatment in the post-enzalutamide population has limited activity (Noonan *et al*, 2013). Similarly ARN-509, which appeared to have greater *in vivo* activity than enzalutamide (Clegg *et al*, 2012), showed indications of diminished activity post-abiraterone compared to the abiraterone-naïve population (Rathkopf *et al*, 2013a). Ultimately, oncologists are faced with the sobering reality of primary resistance to first- and second-line abiraterone or enzalutamide in 20–40% and 60–80% of CRPC patients, respectively. Even those patients that enjoy initial responses will eventually acquire resistance (Scher *et al*, 2010, 2012; de Bono *et al*, 2011).

AR copy number gain and mutation undoubtedly contribute to AR pathway inhibitor resistance [for example, AR gain has recently been linked to lack of response to abiraterone (Carreira *et al*, 2014)], but they cannot explain the entire landscape of progressing disease. It is now widely recognized that the increased transcriptional pressure on the AR gene during CRPC progression can lead to the generation of truncated isoforms of the AR: coding only for the DNA binding and transactivation domains. Since these AR variants (AR-Vs) are missing the LBD, they are constitutively active and impervious to inhibition by conventional AR-targeted agents (Dehm & Tindall, 2011). Their aetiology is complex, since although their genesis frequently lies in aberrant splicing, cell line and xenograft models most dependent on variant expression harbour rearrangements in the AR gene locus that preclude transcription of LBD coding exons (Li *et al*, 2011, 2012). This hard-wired genomic asset uncouples AR-V expression from AR full length (AR_FL_) (Nyquist *et al*, 2013), expediting Darwinian selection. In the clinic, AR-Vs detected in circulating tumour cells from CRPC patients have been recently associated with resistance to abiraterone and enzalutamide (Antonarakis *et al*, 2014). In progressing patients, AR-V expression was always inferior to AR_FL_, preventing rejection of persistent doubts about biological relevance in clinical cohorts. However, with the constant pressure for AR rearrangement and copy gain in castrate conditions, it is simple to imagine partial AR copies coincidentally arising, and if functional, augmenting the activity of AR_FL_ proteins, or possibly even relegating them to passenger status.

Truncated AR-Vs and the rising burden of LBD mutations fortified efforts to develop alternative inhibitors of the AR (Fig1). Compounds targeting the indispensable AR N-terminal and DNA binding domains have shown early promise, reducing AR transcriptional activity in preclinical models (Andersen *et al*, 2010; Myung *et al*, 2013; Dalal *et al*, 2014). However, recent data demonstrated that the N-terminal inhibitor EPI-001 (Andersen *et al*, 2010) has secondary effects on AR activity via modulation of peroxisome proliferator-activated receptor-gamma (PPARγ) (Brand *et al*, 2015), raising questions about the potential to specifically inhibit the intrinsically disordered N-terminal domain. In theory, inhibition of AR mRNA via antisense oligonucleotide (ASO) interference is a simple strategy to reduce expression of all AR species. Although a phase I trial of ASO EZN-4167 in advanced CRPC showed minimal activity and concerning toxicity (Bianchini *et al*, 2013), the drug binds to exon 4, which is missing from the most common AR-Vs. More recently, Yamamoto and colleagues demonstrated that an ASO targeting AR exon 1 (ISIS-ARRx) is sufficient to suppress both AR_FL_ and AR-Vs in enzalutamide-resistant preclinical models (Yamamoto *et al*, 2015). Furthermore, clear anti-tumour activity of ISIS-ARRx in these models provides preclinical support for AR-ASO strategies as a rational third-line approach for AR pathway inhibitor-resistant CRPC.

With abiraterone and enzalutamide now standard of care in first-line CRPC, novel AR-targeted agents, regardless of mechanism of action, will need to show activity in patients progressing on AR pathway inhibitors who will have higher levels of AR expression and aberration.

## Indirect reactivation of the androgen receptor

### Deregulation of androgen receptor co-chaperones

In the absence of ligand in a non-malignant prostate cell, the AR protein forms a complex in the cytoplasm with heat-shock proteins (e.g. HSP70, HSP90) acting as molecular chaperones to maintain the AR in a stable conformation for ligand binding and protect it from proteolysis (Chmelar *et al*, 2007) (Fig1). Ligand binding elicits an AR conformation change, and the receptor is then trafficked to the nucleus (Cano *et al*, 2013). However, the innate stress response induced by castration induces activity of heat-shock proteins and helps insulate the AR axis from degradation (Azad *et al*, 2015c). For example, the stress-induced chaperone, HSP27, is induced by castration and recruited to promote the nuclear transport of the AR (Zoubeidi *et al*, 2007). Overexpression of HSP27 *in vivo* suppresses apoptosis and is sufficient to confer castrate resistance (Rocchi *et al*, 2005; Zoubeidi *et al*, 2010b). Thus, inhibiting HSP90 and/or HSP27 may disrupt the AR foldosome and could sensitize AR-targeted agents.

Indeed, a recent phase II trial of OGX-427 (a second-generation ASO against HSP27) in mCRPC patients demonstrated promising results, doubling the PSA (prostate-specific antigen) response (> 50% decline) rate compared to prednisone alone (Chi *et al*, 2012). Conversely however, phase I/II trials of HSP90 inhibitors in CRPC patients have been disappointing, despite preclinical activity in CRPC models (Heath *et al*, 2008, 2013; Lamoureux *et al*, 2011b; Pacey *et al*, 2011). Alternative targeting strategies are in development, including the use of sulforaphane to inhibit HDAC6 and thereby prevent HSP90 acetylation (Gibbs *et al*, 2009). Overall, the efficacy of HSP90 inhibition is limited by the functional redundancy of molecular chaperones and adaptive feedback mechanisms including the activation of HSF1 [a heat-shock transcription factor that induces, amongst others, HSP70, HSP27 and CLU (clusterin)] (Azad *et al*, 2015c). Since molecular chaperones play key roles in endoplasmic reticulum stress responses and protein homeostasis, co-targeting two or more may overwhelm the ability of cancer cells to regulate their misfolded protein burden. Accordingly, the preclinical activity of HSP90 inhibitors is enhanced via simultaneous targeting of HSF1, HSP27, or CLU (Lamoureux *et al*, 2011a, 2014; Chen *et al*, 2013). Co-targeting the AR simultaneously with HSP90 or HSP27 is also a rational combination strategy: results from phase II trials combining the HSP90 inhibitor AT13387 (NCT01685268) or the HSP27 inhibitor OGX-427 (NCT01681433) with abiraterone will be enlightening (Table1).

### Genomic modification of androgen receptor co-activators

Once in the nucleus, the functions of the AR are mediated by a milieu of co-factors and co-activators capable of modulating the selection and expression of downstream targets (Chmelar *et al*, 2007; Heemers *et al*, 2009; Xu *et al*, 2009). In the context of CRPC, the P160 SRC (steroid receptor co-activator) family genes, NCOA1, NCOA2 and NCOA3 (also known as SRC1-3), have received considerable attention. Overexpression of NCOA1 or NCOA2 can drive increased AR transactivation in castrate conditions (Gregory *et al*, 2001), and depletion of NCOA2 prevents CRPC development in PTEN-deficient mice (Qin *et al*, 2014). Interestingly, NCOA2 up-regulation in CRPC combines genomic and adaptive mechanisms, since it is de-repressed by androgen depletion but also frequently amplified in advanced prostate cancer (Agoulnik *et al*, 2006; Taylor *et al*, 2010). NCOA3 is also linked to prostate cancer cell proliferation and survival (Zhou *et al*, 2005; Yan *et al*, 2008) and is a key target of the ubiquitin ligase SPOP [mutated in 6–15% of prostate cancer (Barbieri *et al*, 2012)]. Wild-type SPOP, but not mutant, promotes ubiquitination (and subsequent degradation) of NCOA3 (Geng *et al*, 2013), and interestingly the AR as well (An *et al*, 2014; Geng *et al*, 2014) (Fig1). Although targeting SPOP in prostate cancer constitutes a considerable challenge, inhibiting downstream proteins escaping ubiquitination in SPOP mutant tumours may be more feasible, lending further credence to strategies targeting the P160 SRC family.

The forkhead protein FOXA1 is a critical interacting partner of the AR, functioning as a pioneer factor to modulate chromatin accessibility and facilitate transcription (Jozwik & Carroll, 2012). In prostate cancer, FOXA1 is capable of specifying unique AR binding sites and has an AR-independent function as a metastasis regulator (Jin *et al*, 2013; Sahu *et al*, 2013). Although it can be genomically amplified, deleted, or mutated in CRPC patients, suggesting complex context-dependent activity (Taylor *et al*, 2010; Barbieri *et al*, 2012; Grasso *et al*, 2012), the precedent set by the development of a FOXM1 inhibitor suggests that forkhead protein modulation in prostate cancer might hold promise (Gormally *et al*, 2014). Interestingly, FOXA1 and AR co-localize on chromatin with GATA2, a transcription factor that enhances recruitment of NCOAs to the AR complex (He *et al*, 2014). Additionally, at the transcriptional level, there appears to be a complex feedback balance between GATA2 and the AR itself, since GATA2 is repressed by the AR and androgen, but is necessary for optimal expression of the AR (He *et al*, 2014). High GATA2 expression predicts poor outcome in prostate cancer patients and further promotes the concept of therapeutically targeting the AR transcriptional complex in CRPC patients. A promising contemporary strategy to disrupt AR in this manner is to use bromodomain inhibitors (e.g. JQ1) to inhibit the chromatin reader BRD4 that interacts with the N-terminal domain of the AR (Asangani *et al*, 2014). Preclinical studies have shown that JQ1 disrupts AR-mediated gene transcription in CRPC models, significantly reducing tumour volume relative to controls (Asangani *et al*, 2014).

An interesting alternative approach to inhibit AR co-activators is to specifically target their interaction with the AR. Recently, potent inhibitors of the AR Binding Factor 3 (BF3) pocket have been developed that demonstrate activity in enzalutamide-resistant preclinical models (Munuganti *et al*, 2014). A novel class of small organic molecules without a peptide backbone (peptidomimetics) have also been recently shown to disrupt AR co-activator interactions and are candidates for clinical development (Ravindranathan *et al*, 2013).

## Adaptive induction of compensatory pathways

The therapeutic targeting of driver aberration (e.g. an overactive AR) can activate adaptive survival pathways, leading to apoptosis inhibition, tumour cell plasticity, and the emergence of treatment resistance (Zoubeidi *et al*, 2010a). Unfortunately, the functional redundancy and heterogeneity of CRPC mean that no single pathway is relevant to all tumours and therefore that therapeutic targeting of a specific pathway is likely of limited benefit. However, combination strategies inhibiting molecules involved in crosstalk between multiple pathways have potential to induce conditional lethality (Carver *et al*, 2011; Azad *et al*, 2015c). Consequently, in concert with AR inhibition, complimentary strategies co-targeting signalling pathways that cooperatively activate the AR, or stress response pathways that maintain homeostasis, represent exciting opportunities to stimulate a high therapeutic index. Only through precise characterization of the stress-induced adaptive response, and the rational development of combinatorial co-targeting strategies, will the full potential of AR pathway inhibition be achieved.

### Activation of kinase-dependent signalling pathways

The profound effect of AR axis inhibition on prostate cancer cells has ramifications for many kinase signalling pathways, particularly those that have accumulated genomic aberration during disease progression. The PI3K/AKT/MTOR pathway is frequently altered in advanced prostate cancer, particularly through deletion of PTEN (> 50% CRPC), but also through mutation (e.g. of PI3KCA) or overexpression of upstream tyrosine kinases (Taylor *et al*, 2010; Grasso *et al*, 2012). PTEN loss leads to greater PI3K activity in castrate conditions and cell proliferation, thereby providing a potential escape route from AR inhibition (Wang *et al*, 2003). In theory, the PI3K/AKT/MTOR pathway marks a putative Achilles heel to target with rational drug design, but it is riddled with functional redundancy and complex compensatory mechanisms. For example, in the absence of PTEN, the AR and PI3K pathways cross-regulate each other via reciprocal feedback, at least in model systems (Carver *et al*, 2011; Mulholland *et al*, 2011). A recent study in breast cancer demonstrated that PTEN loss on the background of PIK3CA mutation actually conferred resistance to a PI3Kα inhibitor (Juric *et al*, 2015). Perhaps unsurprisingly, single agent targeting of the PI3K pathway in CPRC has been singularly underwhelming, with minimal activity in phase II trials of mTOR inhibitors ridaforolimus, temsirolimus and everolimus (Amato *et al*, 2012; Kruczek *et al*, 2013; Templeton *et al*, 2013). Trials combining PI3K and AR inhibition are more promising in terms of preventing compensatory feedback, but may encounter toxicity issues (Thomas *et al*, 2013) (NCT01485861;NCT01634061).

Preclinical data suggest the involvement of other signalling molecules including IGF1, HER2, MET and SRC kinases in the progression of segmented populations after second-line AR pathway inhibition (reviewed in Lorente & De Bono, 2014). However, while kinase-targeted strategies are a successful example of precision therapy for several cancers, the majority fail to produce long-term durable response or complete remission (Zhang *et al*, 2009). Furthermore, tyrosine kinases are rarely altered at the genomic level in CRPC (Grasso *et al*, 2012). As such, it is unlikely that prostate cancer cells are inherently addicted to specific kinase activation (c.f. EGFR mutations in lung cancer) and can instead adapt accordingly to monotherapy inhibition. For example, although up-regulation of HER2 in CRPC results in increased AR transcriptional activity (Mellinghoff *et al*, 2004), lapatinib (a non-selective HER2 inhibitor) showed very limited activity in CRPC patients (Whang *et al*, 2013). Other tyrosine kinase inhibitors targeting MET (cabozantinib), VEGF (sunitinib), endothelin (atrasentan, zibotentan) and SRC (dasatinib) failed in phase III trials to improve overall survival of post-docetaxel CRPC patients (Lorente & De Bono, 2014; Michaelson *et al*, 2014; Sridhar *et al*, 2014). These failures are likely driven by the aforementioned lack of absolute kinase dependency in CRPC, and subsequent bypass via compensatory pathways. A non-ideal choice of co-targeting drug (e.g. docetaxel) may also have played a role. More promisingly, there are several ongoing phase I/II trials evaluating tyrosine kinase inhibitors in combination with abiraterone or enzalutamide, and it is conceivable that eliminating context-dependent tyrosine kinase activation as a compensatory mechanism for AR inactivation will enhance the efficacy of AR-targeted agents (Table1).

### Activation of stress response pathways

Cellular stress can drive the evolution and adaptation of cancer cells. The stress response that is activated by castration in AR-driven prostate cancers includes up-regulation of molecular chaperones that regulate protein homeostasis and diverse survival signalling and transcriptional survival networks (Garrido *et al*, 2006; Dai *et al*, 2007; Zoubeidi & Gleave, 2012; Matsumoto *et al*, 2013). It is unsurprising therefore that certain chaperones are frequently up-regulated in prostate and other cancers and their expression correlates with metastases, treatment resistance, and poor survival (Azad *et al*, 2015c). Outside of the AR signalling axis (that is insulated by the heat-shock proteins discussed above), CLU is the most credentialed molecular chaperone capable of driving treatment resistance in CRPC cells. CLU is induced by stress-activated transcription factors, including EGR1, HSF1 and YBX1, to constrain apoptosis through inhibition of activated Bax and suppression of protein aggregation via autophagy activation (Zoubeidi & Gleave, 2012; Zhang *et al*, 2014). Accordingly, CLU inhibition potentiates activity of anti-cancer therapeutics in preclinical models (Sowery *et al*, 2008; Zoubeidi *et al*, 2010a).

Since CLU is challenging to target with traditional small-molecule inhibitors, the ASO drug OGX-011 (custirsen) was developed to instead inhibit mRNA translation. A phase II trial reported a 7-month overall survival benefit of OGX-011 in combination with docetaxel, compared to docetaxel alone, in chemotherapy-naïve mCRPC (Chi *et al*, 2010), leading to the initiation of randomized phase III studies: SYNERGY (NCT01188187) and AFFINITY (NCT01578655). SYNERGY randomized 1,022 men with mCRPC to OGX-011 in combination with docetaxel and prednisone to docetaxel and prednisone alone. Survival results were first presented at ESMO in 2014 (Chi *et al*, 2014) and indicated that addition of OGX-011 did not meet the primary endpoint of a statistically significant improvement in overall survival in men with CRPC compared to docetaxel/prednisone alone (median survival 23.4 versus 22.2 months, respectively; hazard ratio 0.93 and *P*-value 0.207). AFFINITY, which assesses the second-line indication comparing cabazitaxel with or without OGX-011 in post-docetaxel-treated CRPC, has completed enrolment and should read out by end of 2015 (Table1). The failure of OGX-011 in SYNERGY, despite robust preclinical proof-of-principle, phase I on-target suppression data (Chi *et al*, 2005), and phase II survival signals (Chi *et al*, 2010), illustrates the challenge of selecting appropriate combinations based on phase II signals, and how changes in treatment landscape (in this case the approval of abiraterone and enzalutamide) mid-development may alter outcomes. Given its location on chromosome 8p proximal to the prostate cancer tumour suppressor gene NKX3-1, the CLU gene is homozygously deleted in ∼20% of CRPC patients (Grasso *et al*, 2012), a population that likely confounded OGX-011 evaluation.

### Somatic deregulation to DNA repair and cell cycle machinery

At diagnosis, the genomic landscape of prostate cancer is very heterogeneous, bearing a heavy burden of genomic rearrangement replete with rare combinations of tumour suppressor inactivation and oncogene activation (Taylor *et al*, 2010; Barbieri *et al*, 2012; Grasso *et al*, 2012; Baca *et al*, 2013; Wyatt *et al*, 2014). Furthermore, since the AR program regulates the transcriptional programs of DNA repair genes in prostate cancer cells, AR axis inhibition has the potentially undesirable side-effect of promoting genomic instability (Polkinghorn *et al*, 2013). In theory however, as the life-expectancy of contemporary CRPC patients rises, there is increasing opportunity for accumulating genomic aberration to render certain cellular functions useless. For example, it is now recognized that 12% of advanced prostate cancers have accrued defects to DNA mismatch repair machinery and have consequently developed a “hypermutated” genotype similar to that observed in microsatellite instability in colon cancer (Pritchard *et al*, 2014).

The shedding of specific cellular machinery can remove aspects of functional redundancy that shields cancer cells from monotherapy. Homozygous somatic aberration to key mediators of homologous recombination in DNA repair, including BRCA2 and ATM, now appears common in advanced CRPC (Grasso *et al*, 2012). This discovery proposed the intriguing hypothesis that targeting DNA repair machinery via poly (ADP-ribose) polymerase (PARP) inhibition will induce synthetic lethality exclusive to tumour cells. PARP1 is a particularly attractive target in CRPC since it also plays a key role supporting both AR function (Schiewer *et al*, 2012b) and ETS transcription factor activity (Brenner *et al*, 2011). Remarkably, durable responses have been reported for CRPC patients treated with niraparib, a PARP1 and PARP2 inhibitor (Sandhu *et al*, 2013). Considerable optimism surrounds the current phase II evaluation of olaparib (a selective PARP1 targeted agent) as a monotherapy in CRPC (NCT01682772), and a phase II trial of veliparib (a PARP1 and PARP2 inhibitor) in combination with abiraterone and prednisone (NCT01576172).

In prostate cancer, the AR facilitates cell proliferation via effects on the cyclin/cyclin-dependent kinase (CDK)/retinoblastoma (RB) pathway (Schiewer *et al*, 2012a). This critical cell cycle machinery is frequently deregulated in CRPC, with CCND1 amplification and/or RB1 loss the most recognized genomic contribution, detectable in 5–8 and 30% of CRPC, respectively (Bubendorf *et al*, 1999; Grasso *et al*, 2012). In tumours with functional RB1, inhibition of CDK4 and CDK6 activity has a significant suppressive effect on cell proliferation (Comstock *et al*, 2013). The future success of trials evaluating CDK4/6 inhibitors in CRPC is likely to hinge on enrichment with patients whose tumours exhibit cyclin/CDK activation in the background of intact RB1. Conversely, tumours with complete RB1 loss have hard-wired activation of cyclin/CDK-mediated cell proliferation. Although this renders CDK4/6 inhibition redundant, cells are presumably less able to modulate proliferation. In apparent support of this concept, recent evidence suggests that RB1-depleted tumours are more sensitive to the novel taxane chemotherapy cabazitaxel (de Leeuw *et al*, 2014, 2015), which was recently approved for patients progressing on docetaxel after demonstrating a survival benefit over mitoxantrone and prednisone (de Bono *et al*, 2010). Importantly, cabazitaxel does not appear to exhibit cross-resistance with AR-targeted agents, suggesting effects are independent of the AR pathway (Al Nakouzi *et al*, 2014; Pezaro *et al*, 2014b; van Soest *et al*, 2014; de Leeuw *et al*, 2015). Genomic mechanisms of resistance to taxane-based chemotherapy are unclear, although recent data suggest that ERG affects microtubule dynamics and that ERG overexpressing tumours are consequently more resistant to docetaxel (Galletti *et al*, 2014). Interestingly, ERG itself represents a potential therapeutic target in tumours with ERG rearrangements (Wang *et al*, 2014). Since the majority of rearrangements place ERG under direct control of an AR-regulated promoter (e.g. from the TMPRSS2 gene), ERG is overexpressed as a direct consequence of AR reactivation in CRPC (Cai *et al*, 2009). Although the clinical development of specific ERG inhibitors has proved elusive to date, a recent study showed that inhibition of the ERG-stabilizing deubiquitinase USP9X results in ERG depletion and may represent a potential therapeutic strategy (Wang *et al*, 2014).

## The protective role of the microenvironment

Cancer cells reside within a complex microenvironment that can either compromise or augment survival and growth (Sun & Nelson, 2012). Furthermore, as prostate cancer switches from an endocrine-driven disease to a paracrine- or autocrine-driven disease after CRPC development, tumour cells become increasingly reliant on the microenvironment for survival. For example, prostate-cancer-associated stromal cells can facilitate androgen biosynthesis in tumour cells under castrate conditions (Arnold *et al*, 2008; Mizokami *et al*, 2009; Sillat *et al*, 2009). More recently, an elegant study demonstrated that in the aftermath of genotoxic therapy, the innate DNA damage response in benign stromal cells stimulates secretion of cytokines, growth factors and proteases that ultimately promote therapy resistance in tumour cells (Sun *et al*, 2012).

Strategies designed to target the tumour microenvironment are attractive, not least since normal cells cannot easily evolve to a resistant state. The most effective approach in many solid malignancies has been to interfere with VEGF-mediated blood vessel recruitment to tumour tissue. Unfortunately, attempts to repurpose anti-angiogenic drugs for CRPC have failed. For example, the VEGF-targeted agents bevacizumab, aflibercept and lenalidomide all failed to improve the overall survival conferred by docetaxel in large phase III trials (Kelly *et al*, 2012; Petrylak *et al*, 2012; Tannock *et al*, 2013), collectively implying that VEGF-mediated angiogenesis is not the sole driver of progression in bone-predominant mCRPC. Similarly, endothelin receptor (END1) targeting agents, atrasentan and zibotentan, also failed in phase III studies (Carducci *et al*, 2007; Nelson *et al*, 2012), despite biologic and preclinical proof-of-principle as well as signals of activity in phase II studies. Overall, the development of these angiogenesis inhibitors was challenged by lack of single agent activity that compromised detection and/or interpretation of robust activity signals. In the case of zibotentan, a randomized phase II versus placebo in men with M1 CRPC demonstrated improved markers of bone turnover and initially signalled significantly improved survival but with maturation this benefit disappeared (James *et al*, 2009, 2010). Based on the initial survival benefit, a phase III trial enrolled 594 patients, but survival was not significantly prolonged, in part due to insufficient sample size.

A more successful strategy has been to exploit the remarkable propensity of tumour cells to form metastatic deposits in the bone. Despite decades of availability, crude radiopharmaceuticals have demonstrated only limited uptake due to incidental bone marrow toxicity from errant beta-particles. However, in 2013, the calcium mimetic radium-223 dichloride (Xofigo) was approved for the treatment of bone metastatic CRPC (Parker *et al*, 2013). Activity is reliant on the potent effect, but short range of alpha radiation emitted from radium-223 decay: reducing peripheral damage to healthy tissue while maintaining powerful anti-tumour efficacy. Overall, although radiopharmaceuticals do not strictly target the microenvironment, their rational use has demonstrated that it is possible to elicit overall survival gains by selectively targeting the bone niche.

Transient cell populations that migrate in and out of the ecosystem can also influence tumour dynamics. Tumours arise in an immunocompetent environment, interacting with innate and adaptive branches of the host immune system (May *et al*, 2011). Although the host immune system is capable of mounting an anti-tumour response, tumour cells frequently enjoy an excess of regulatory and suppressor T cells, blunting the effector response. In the apoptotic aftermath of initial androgen deprivation therapy, leucocytes are further recruited to tumour tissue, but rather than reacting to the cancer, they may promote progression to CRPC (Luo *et al*, 2007; Ammirante *et al*, 2010). The field of immunotherapeutics seeks to exploit the potent and intact anti-tumour response and is reviewed in-depth elsewhere (Madan *et al*, 2013; Makkouk & Weiner, 2015; May *et al*, 2011). The most advanced clinical strategies for CRPC are therapeutic vaccines that induce a novel anti-tumour response, and immune checkpoint modulators that prevent suppression of the existing response. Sipuleucel-T (provenge) is a therapeutic vaccine generated by *ex vivo* stimulation of antigen presenting cells. It became the first immunotherapy approved for use in prostate cancer after demonstrating a significant overall survival benefit in asymptomatic or minimally symptomatic mCRPC (Kantoff *et al*, 2010a). Similarly, the vector-based vaccine PSA-TRICOM (PROSTVAC), which generates an *in vivo* response against cells expressing PSA, showed an overall survival benefit for mCRPC patients in phase II trials (Gulley *et al*, 2010; Kantoff *et al*, 2010b) and is currently under phase III evaluation in men with asymptomatic or minimally symptomatic mCRPC (NCT01322490). Ipilimumab (yervoy) is an antibody that binds to CTLA-4 and prevents the suppression of cytotoxic T cells, resulting in a more aggressive anti-tumour immune response (Slovin *et al*, 2013). Although a phase III trial of ipilimunab in mCRPC did not show a significant gain in overall survival (Kwon *et al*, 2014), there was evidence of activity in patients with favourable prognoses (Drake *et al*, 2014). This is particularly relevant given that patients with the least aggressive disease appear to receive the most benefit from sipuleucel-T and PSA-TRICOM.

Despite overall survival gains, immunotherapeutics have not demonstrated impact on short-term progression. Rather, preliminary analyses suggest their efficacy in prostate cancer is via long-term alterations in tumour growth kinetics (Beer *et al*, 2011; Gulley *et al*, 2013), potentially explaining the observations of increased activity in less advanced disease. Furthermore, since an anti-tumour response tends to be sustained and can even evolve over time to target more antigens [known as antigen cascade (Disis *et al*, 2004)], there is a strong rationale to evaluate immunotherapies earlier in disease progression. Combining vaccines with checkpoint inhibition may also enhance the anti-tumour response and is currently being evaluated (Jochems *et al*, 2014) (NCT01832870).

## Inactivation of the androgen receptor

The continual accrual of genomic aberration together with the de-differentiating force of sustained AR inhibition provides opportunities for tumour cells to escape dependence on AR signalling. One potential escape route is via up-regulation of compensatory steroid receptors that show high homology to the AR, suggesting a degree of functional redundancy. Indeed, oestrogen receptor (ER) alpha and beta are frequently upregulated in advanced prostate cancer, but whereas preclinical data support the use of ER targeted agents, there is no evidence of clinical response to ER modulation in CRPC patients (Nelson *et al*, 2014). A recent study demonstrated that the glucocorticoid receptor (GR) can regulate a proportion of the AR cistrome and its up-regulation in AR-repressed conditions may represent a mechanism to re-initiate mitogenesis in CRPC (Arora *et al*, 2013; Sahu *et al*, 2013).

Tumours can also evolve or adapt to become a completely AR-independent disease. In the contemporary disease setting of potent AR targeting, it has become common to observe progression of advanced CRPC in the absence of high serum PSA (a marker of AR activity) and with atypical visceral metastases (Beltran *et al*, 2012; Aparicio *et al*, 2013; Pezaro *et al*, 2014a). Predictably, AR-independent prostate cancer is highly heterogeneous, but a major established subtype is neuroendocrine prostate cancer (NEPC) (Beltran *et al*, 2011; Epstein *et al*, 2014). Typical NEPC expresses a dominant and irreversible neuronal phenotype (Lin *et al*, 2014), which complicates attempts to delineate malignant (and therefore targetable) aspects of the disease. Importantly however, since the AR is either incidental to growth or no longer expressed at all, conventional CRPC therapies are redundant and platinum-based chemotherapy are effective, although relapse is rapid and overall survival remains poor (Aparicio *et al*, 2013).

**Figure 2:**
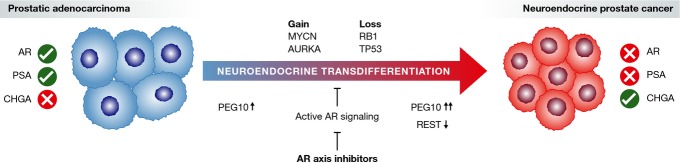
Neuroendocrine transdifferentiation in response to AR axis inhibition Illustration of the adaptive response to AR axis inhibition that can result in prostatic adenocarcinoma transforming to neuroendocrine prostate cancer. Typical disease markers including chromogranin A (CHGA) are shown at either end of the diagram. Genomic aberration thought to facilitate transdifferentiation is indicated above the arrow. Small arrows indicate up- and down-regulation, respectively.

A large body of evidence suggests that NEPC arises from prostatic adenocarcinoma cells via an adaptive process termed “neuroendocrine transdifferentiation” (Guo *et al*, 2011; Lotan *et al*, 2011; Williamson *et al*, 2011; Lin *et al*, 2014). However, even in the contemporary setting, less than a quarter of patients harbour NEPC foci at death, implying that only certain tumours are capable of attaining a proliferative NEPC state. Although prostatic adenocarcinoma cells are adapted to their primary niche, they are presumably poorly adapted to a neuronal niche that utilizes distinct mechanisms of innate tumour suppression. Therefore, to attain a proliferative NEPC tumour, additional and specific genomic perturbation to neuronal tumour suppressors and/or oncogenes is likely required (Fig2). Emerging data suggest these predisposing aberrations include loss of RB1 and TP53, and gain of MYCN and AURKA (Beltran *et al*, 2011; Chen *et al*, 2012; Tan *et al*, 2014). Although identification of the latter led to initiation of a phase II trial of the AURKA inhibitor MLN8237 in NEPC (NCT01799278), there have been few novel leads for targeting this lethal disease variant, partly due to a paucity in preclinical models. Recently, a first-in-field patient-derived xenograft model of neuroendocrine transdifferentiation has been described (Lin *et al*, 2014). Molecular characterization of this model led to the discovery that PEG10, a placental gene, is de-repressed during the adaptive response to AR inhibition and highly up-regulated in clinical NEPC (Akamatsu *et al*, 2014). PEG10 is regulated by the AR and promotes growth and invasion of cancer cells in the context of RB1 and TP53 loss. Furthermore, since expression of PEG10 in adult tissue is extremely limited, it represents a strong candidate for therapeutic targeting. Discoveries such as AURKA, MYCN and PEG10 are likely to be the first of in a wave of mechanistic insights into the development of NEPC, and their critical role during progression suggests that the optimal strategy for select patients may rely on intervening prior to transformation to NEPC.

## Future directions

It is plausible that the existing armamentarium of novel agents can elicit greater anti-tumour responses when used at earlier stages in disease, or in rational combination with each other. Indeed, strong responses have been observed after neo-adjuvant or front-line enzalutamide or abiraterone administration. Trials combining these two agents will also prove informative (NCT01650194, NCT00268476). However, genomic biomarkers of response and resistance emerging from prospective studies must be integrated into clinical trial design in order to improve chances of success. As described in this review, in the aftermath of cytotoxic and potent second-line AR-targeted agents, the CRPC landscape is riddled with aggressive, heterogeneous and adaptive clones. Therefore, to facilitate trial enrichment or optimal drug sequencing, patient tumours must be monitored for novel molecular changes. Unfortunately, logistical difficulties and significant morbidity have long precluded routine collection of mCRPC tissue biopsies. Even where possible, one is faced with tumour cellularity issues (particularly in bone metastases), and under-sampling of a single site amongst a highly heterogeneous ecosystem of metastases. Although remarkable efforts from Prostate Cancer Foundation/Stand Up To Cancer Dream Teams have established robust protocols for mCRPC tissue collection, the likelihood for widespread adoption is low, and a more minimally invasive strategy is urgently required.

**Figure 3:**
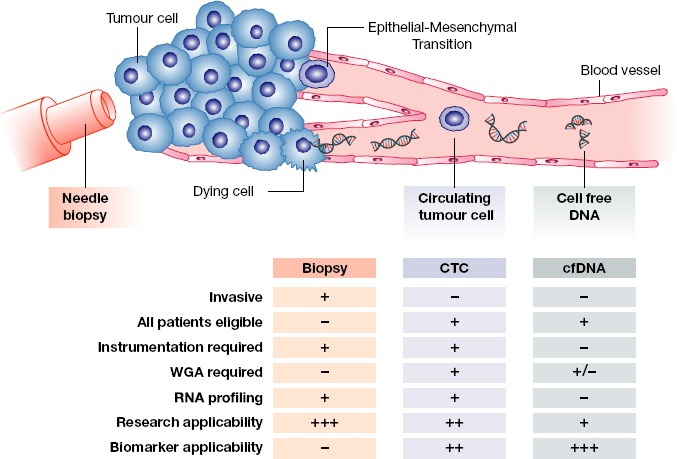
Applicability of the liquid biopsy for CRPC Schematic showing the relative strengths and weaknesses of a tumour tissue biopsy, circulating tumour cell analysis, and cell-free DNA analysis for monitoring patients with CRPC. WGA = whole-genome amplification.

Since tumour material is continually shed into the bloodstream, liquid biopsy (extensively reviewed in Crowley *et al*, 2013; Diaz & Bardelli, 2014; Heitzer *et al*, 2013; Joosse *et al*, 2014) holds great promise for improving CRPC patient management. From a trial enrichment/biomarker perspective perhaps the most exciting developments have emerged from studies of tumour-derived cell-free DNA (cfDNA) extracted from patient plasma. Compared to circulating tumour cells (CTCs), cfDNA analyses provide a global survey of tumour status, and the recent application of targeted sequencing to cfDNA extracted temporally from 16 advanced prostate cancer patients was able to accurately monitor the dynamics of lethal tumour clones (Carreira *et al*, 2014). Allowing for the compromising dilution effect of “normal” cfDNA, relatively simple next-generation sequencing approaches are sufficient to robustly detect amplifications and mutations, making cfDNA analyses ideal for monitoring AR status longitudinally in CRPC patients. Indeed, a recent study used combination of copy number profiling and next-generation sequencing to identify AR amplifications and mutations in the cfDNA of mCRPC patients progressing on novel systemic agents (Azad *et al*, 2015b). Interestingly, AR alterations accompanied enzalutamide resistance, and the presence of pre-treatment AR amplifications or mutations were predictive for adverse outcomes on enzalutamide (Azad *et al*, 2015b; Gleave & Chi, 2015). CTCs will remain critical for basic and clinical research alike, due to the ability to profile the transcriptome (e.g. for truncated AR variants) and the potential of establishing cultures. However, the broad utility of cfDNA (Fig3) means that it will likely assume a key role alongside CTCs in clinical trial design and CRPC patient management.

For CRPC patients, the story of recent years has been one of tempered success, with overall survival gains afforded by novel systemic agents countered by inevitable resistance and emergence of a more aggressive and heterogeneous disease. Importantly however, the conceptual mechanisms underpinning progression to initial CRPC appear to be equally relevant in the second- and third-line resistance setting. In the future, the optimal anti-tumour strategies will be those that target rational combinations of pathways in concert with AR, and select appropriate patient populations within which to test.

**Pending issues**

- Can we develop treatment predictive biomarkers that will allow optimal drug sequencing strategies tailored to individual patients?
- How can we develop therapies for patients with AR-negative CRPC?
- Will the new wave of novel AR-targeted therapies for CRPC prove even more effective when moved earlier in disease progression?

## Abbreviations

Adaptive stress response: A mechanism by which cells can induce the expression of protective proteins to prevent cell damage
Androgen receptor (AR) antagonists: Compounds that compete with endogenous steroid hormones to bind the AR and thereby inhibit protein function
AR variants: Shortened forms of the AR protein that are missing the ligand-binding domain and therefore do not require activation by steroid molecules
Castration-resistant prostate cancer (CRPC): Progression of prostate cancer to a form that can grow in very low levels of circulating testosterone
Cross-resistance: Tolerance of cancer cells to a normally effective drug due to prior resistance to a drug with a similar mechanism of action
Immunotherapy: Drugs that induce or enhance the body's anti-tumour immune response
Liquid biopsy: Non-invasive blood tests that detect tumour cells or pieces of tumour DNA that are circulating in the blood
Steroidogenesis: Enzymatic process by which cholesterol is converted to steroid hormones
Tumour heterogeneity: Different tumour cells within a single patient can show different genotypic and phenotypic profiles
Tumour microenvironment: The normal cells that surround tumour cells and provide them with nutrients and support
